# Factors associated with hospitalization for community-acquired pneumonia in home health care patients in Taiwan

**DOI:** 10.1007/s40520-019-01169-8

**Published:** 2019-03-14

**Authors:** Chien-Ju Lin, Yu-Chen Chang, Meng-Ting Tsou, Hsin-Lung Chan, Ying-Ju Chen, Lee-Ching Hwang

**Affiliations:** 1 The Department of Family Medicine, MacKay Memorial Hospital, No. 92, Sec. 2, Zhongshan N. Rd., Zhongshan Dist., Taipei, 104 Taiwan, ROC; 2 The Telehealth and Home Care Center, MacKay Memorial Hospital, No. 92, Sec. 2, Zhongshan N. Rd., Zhongshan Dist., Taipei, 104 Taiwan, ROC; 3 The Department of Medicine, MacKay Medical College, No. 46, Sec. 3, Zhongzheng Rd., Sanzhi Dist, 252 New Taipei City, Taiwan, ROC

**Keywords:** Pneumonia, Home health care, Risk factors, Malnutrition, Nasogastric tube

## Abstract

**Background:**

Pneumonia is a leading cause of hospitalization and death worldwide. However, studies focusing on risk factors of community-acquired pneumonia (CAP) in the home health care (HHC) population remain scarce.

**Aims:**

This study aimed to evaluate risk factors associated with hospitalization for CAP among HHC patients in Taiwan.

**Methods:**

This retrospective cross-sectional study extracted data from patients’ electronic medical records between 1 January 2017 and 31 December 2017. Multiple logistic regression analyses were performed to explore factors associated with hospitalization for CAP.

**Results:**

In total, 598 patients (men/women: 236/362) were included. One hundred ninety-nine patients (33.28%) were hospitalized for pneumonia. Inpatients showed a higher proportion of the following: male sex, functional impairment, hypoalbuminemia, anemia, nasogastric tube use, excessive polypharmacy, stroke, dementia, heart failure, chronic respiratory disease, and chronic liver disease. Furthermore, nasogastric tube use (odds ratio [OR] 3.01, 95% confidence interval [CI] 1.88–4.82), anemia (OR 2.37, 95% CI 1.48–3.80), male sex (OR 2.14, 95% CI 1.43–3.20), chronic respiratory disease (OR 2.09, 95% CI 1.33–3.30), dementia (OR 1.94, 95% CI 1.27–2.97), heart failure (OR 1.69, 95% CI 1.11–2.56), and hypoalbuminemia (OR 1.57, 95% CI 1.03–2.40) significantly increased the risk of hospitalization for CAP.

**Conclusions:**

Our results revealed risk factors associated with hospitalization for CAP in HHC patients. In addition to chronic diseases, malnutrition is an important risk factor. Caregivers should make prompt assessments and take preventive measures for such patients.

## Background

Pneumonia is a common cause of morbidity and mortality in adults worldwide. The overall rate of community-acquired pneumonia (CAP) is approximately 5.16–7.06 cases per 1000 persons per year. Overall mortality varies between 7.3 and 13.3% and is especially higher in patients who require hospitalization [1, 2]. Identifying and modifying risk factors are of paramount importance for reducing pneumonia-related death. However, previous studies have mostly explored risk factors among the general population or residents in long-term care facilities [3, 4].

Compared with general population, home health care (HHC) patients may have more comorbid conditions and greater functional impairment. Unlike hospitals or long-term care facilities, the patient’s home environment usually lacks sufficient spaces or resources. Additionally, HHC patients are cared by their family members or care workers without formal training. These situations pose special challenges and potential hazards, which put HHC patients at risk for infections. In HHC setting, most published studies evaluating the incidence and risk factors of infection focused primarily on patients receiving home parenteral nutrition (HPN) treatment. In contrast, few studies have examined infection rates in the general HHC population; these studies found that 5.1–11.5% of HHC patients had at least one episode of infection during their service period [5, 6]. The most common causes were pneumonia, urinary tract infection, and cellulitis. The most frequently reported risk factor for nonspecific infection was indwelling devices [5, 6]. Another study found that approximately 3.5% of HHC patients developed infections that required emergency care treatment or hospitalization [7]. Although many studies recognized underlying diseases related to risks of infection, the reported medical conditions varied largely among studies [5–7].

In Taiwan, the National Health Insurance (NHI) system has provided HHC since 1995. According to the NHI program, a patient qualified for HHC needs to fulfill the following conditions: (1) limited ability of self-care (ECOG performance grade 3 or more or Barthel index score 60 or less), and (2) definite medical or nursing care needs. One study revealed the most common reasons for hospitalization in HHC patients were pneumonia and urinary tract infection. The admission rates correlated positively with multi-morbidities [8]. However, to our knowledge, published literature regarding CAP in the HHC population is limited. We, therefore, conducted this study to evaluate risk factors associated with hospitalization for CAP in HHC patients.

## Methods

### Participants

This retrospective study was conducted in the home health care unit of a medical center in northern Taiwan and approved by the Institutional Review Board of MacKay Memorial Hospital. Data were collected from 1 January 2017 to 31 December 2017. Patients aged ≥ 18 years and who were receiving home care for at least 3 months were included. We reviewed medical records, including home visit records, outpatient department records, admission notes, discharge summaries, and laboratory data. We excluded patients whose relevant data were insufficient.

### Measurement and data collection

From the medical records, the following details were extracted: age, sex, caregiver, smoking history, Barthel index scores, seasonal influenza vaccination history, and use of nasogastric or tracheostomy tube. We calculated mean body mass index, mean hemoglobin value, and mean serum albumin level during the study period as markers of nutritional status [9]. Body mass index (BMI) was calculated based on estimated body weight and height using the formula for Chinese adults [10, 11]. Anemia was defined as hemoglobin level < 12 g/dL and 13 g/dL in women and men, respectively, according to World Health Organization criteria. Hypoalbuminemia was defined as serum albumin level < 3.5 g/dL.

Comorbidities were documented based on the International Classification of Diseases, Tenth Revision, Clinical Modification (ICD-10-CM) codes used during the pneumonia hospitalization and in medical records prior to admission. Comorbidities included stroke, dementia, Parkinson’s disease, diabetes, heart failure, chronic respiratory disease (asthma, chronic obstructive pulmonary disease, and chronic respiratory failure), chronic kidney disease, chronic liver disease (chronic hepatitis, liver cirrhosis, alcoholic liver disease, and chronic hepatic failure), and malignancy. We also recorded the amount of prescribed medications for chronic diseases. Excessive polypharmacy was defined as the prescription of ≥ 10 daily drugs [12].

Pneumonia is caused by bacteria, viruses, or other organisms; however, it can be difficult to distinguish which is the cause of a specific infection by clinical features. Expectorated sputum is not always reliable because results of sputum culture rely heavily on good sample collection. In our study, viral testing, including rapid influenza test, was not routinely obtained from all patients. Therefore, all causes of CAP were considered in our study. Hospitalization for pneumonia was identified from admission and discharge codes according to ICD-10-CM (J10.0 influenza with pneumonia, other influenza virus identified; J11.0 influenza with pneumonia, virus not identified; J12 viral pneumonia; J13 pneumonia due to *Streptococcus pneumoniae*; J14 pneumonia due to *Haemophilus influenzae*; J15 bacterial pneumonia; J16 pneumonia due to other infectious organisms; J17 pneumonia in diseases classified elsewhere; J18 pneumonia, organism unspecified). Diagnosis was further confirmed based on the radiology reports.

Patients diagnosed with hospital-acquired pneumonia were excluded. For patients with repeated episodes of pneumonia requiring admission in the study period, we included only the first episode for the analysis.

### Statistical analysis

SPSS version 22.0 (SPSS INC, Chicago, IL, USA) was used for all statistical analyses. Student’s *t* test and the Chi square test were performed to assess differences between groups. Multiple logistic regression analyses were performed to identify factors associated with hospitalization for pneumonia. Odds ratios (ORs) with 95% confidence intervals (95% CIs) were calculated. A two-tailed *p* value < 0.05 was considered statistically significant.

## Results

A total of 598 HHC patients were included for the statistical analysis (men/women: 236/362). The average age of the patients was 81.9 ± 11.3 years. Most patients were totally functionally dependent (Barthel index scores of 0–20). The patients were cared for mostly by their family members (53.2%), followed by foreign care workers (44.3%) and domestic care workers (2.5%). Anemia was detected in the majority of the study population (73.2%), and hypoalbuminemia was found in 160 patients (26.8%). Stroke appeared to be the most common comorbidity with a prevalence surpassing 50%; nearly two-thirds of patients used a nasogastric tube. Eighty-five (14.2%) patients had a smoking history, and 74 of them were men. The characteristics of the study sample are summarized in Table 1.

**Table 1:** Basic characteristics of the study population

	*n* (%) or mean ± SD
Age (years)	81.9 ± 11.3
Sex (female, *n* %)	362 (60.5)
Caregiver	
Family member	318 (53.2)
Domestic care worker	15 (2.5)
Foreign care worker	265 (44.3)
Past smoking history	85 (14.2)
Barthel index scores	10.3 ± 13.3
Total dependency (0–20 points)	489 (81.8)
Severe dependency (21–60 points)	109 (18.2)
Received seasonal influenza vaccine	159 (26.6)
Nutritional assessment	
BMI (kg/m^2^)	22.0 ± 3.6
Hypoalbuminemia (albumin < 3.5 g/dL)	160 (26.8)
Anemia	438 (73.2)
Nasogastric tube use	379 (63.4)
Tracheostomy tube use	39 (6.5)
Excessive polypharmacy (taking ≥ 10 medications)	125 (20.9)
Comorbidities	
Stroke	301 (50.3)
Dementia	185 (30.9)
Parkinson’s disease	98 (16.4)
Diabetes	278 (46.5)
Heart failure	208 (34.8)
Chronic respiratory disease	123 (20.6)
Chronic kidney disease	267 (44.6)
Chronic liver disease	260 (43.5)
Malignancy	97 (16.2)

*SD* standard deviation, *BMI* body mass index

One hundred and ninety-nine patients (33.3%) were admitted for pneumonia, 23 (11.6%) of whom died during that admission. Almost half (48.9%) of the survivors were readmitted for pneumonia at least once during the study period. As presented in Table 2, inpatients showed a higher proportion of the following conditions: male sex, lower Barthel index scores, excessive polypharmacy, and nasogastric tube use. In addition, stroke, dementia, heart failure, chronic respiratory disease, chronic liver disease, anemia, and hypoalbuminemia were more prevalent in the hospitalized group than in the control group.

**Table 2:** Differences in characteristics and comorbidities of the study population

Variable^a^*N* (%)	Non-hospitalization for pneumonia (*n* = 399)	Hospitalization for pneumonia (*n* = 199)	*p* value
Age (years)	81.6 ± 12.0	82.7 ± 9.7	0.221
Sex			< 0.0001
Male	136 (34.1)	100 (50.2)	
Female	263 (65.9)	99 (49.8)	
Past smoking history	50 (12.5)	35 (17.6)	0.095
Caregiver			0.298
Family member	221 (55.4)	97 (48.7)	
Domestic care worker	10 (2.5)	5 (2.5)	
Foreign care worker	168 (42.1)	97 (48.7)	
Barthel index scores			< 0.0001
Total dependency (0–20 points)	307 (76.9)	182 (91.5)	
Severe dependency (21–60 points)	92 (23.1)	17 (8.5)	
Received seasonal influenza vaccine	110 (27.6)	49 (24.6)	0.442
Nutritional assessment			
BMI (kg/m^2^)	22.2 ± 3.7	21.8 ± 3.5	0.251
Hypoalbuminemia (albumin < 3.5 g/dL)	92 (23.1)	68 (34.2)	0.004
Anemia	272 (68.2)	166 (83.4)	< 0.0001
Nasogastric tube use	217 (54.4)	162 (81.4)	< 0.0001
Tracheostomy tube use	23 (59.2)	16 (8.0)	0.288
Excessive polypharmacy (taking ≥ 10 medications)	71 (17.8)	54 (27.1)	0.008
Comorbidities			
Stroke	189 (47.4)	112 (56.3)	0.04
Dementia	106 (26.6)	79 (39.7)	0.001
Parkinson’s disease	62 (42.1)	36 (18.1)	0.427
Diabetes	181 (45.4)	97 (48.7)	0.435
Heart failure	122 (30.6)	86 (43.2)	0.002
Chronic respiratory disease	62 (15.5)	61 (30.7)	< 0.0001
Chronic kidney disease	177 (44.4)	90 (45.2)	0.841
Chronic liver disease	162 (40.6)	98 (49.3)	0.044
Malignancy	64 (16.0)	33 (16.6)	0.865

*SD* standard deviation, *BMI* body mass index

^a^Data are presented as number (percent) or mean ± SD

In the multiple logistic regression model, nasogastric tube use (odds ratio [OR] 3.01, 95% confidence interval [CI] 1.88–4.82), anemia (OR 2.37, 95% CI 1.48–3.80), male sex (OR 2.14, 95% CI 1.43–3.20), chronic respiratory disease (OR 2.09, 95% CI 1.33–3.30), dementia (OR 1.94, 95% CI 1.27–2.97), heart failure (OR 1.69, 95% CI 1.11–2.56), and hypoalbuminemia (OR 1.57, 95% CI 1.03–2.40) significantly increased the risk of hospitalization for CAP after adjusting for potential confounders as shown in Table 3.

**Table 3:** Multiple logistic regression analysis of factors associated with hospitalization for pneumonia in home health care patients

Variable	OR (95% CI)	*p* value
Sex, male	2.14 (1.43–3.20)	< 0.0001
Totally functionally dependent (Barthel index scores of 0–20)	1.67 (0.87–3.20)	0.121
Hypoalbuminemia (albumin < 3.5 g/dL)	1.57 (1.03–2.40)	0.035
Anemia	2.37 (1.48–3.80)	< 0.0001
Nasogastric tube use	3.01 (1.88–4.82)	< 0.0001
Excessive polypharmacy (taking ≥ 10 medications)	1.49 (0.94–2.37)	0.092
Stroke	1.29 (0.87–1.90)	0.204
Dementia	1.94 (1.27–2.97)	0.002
Heart failure	1.69 (1.11–2.56)	0.014
Chronic respiratory disease	2.09 (1.33–3.30)	0.002
Chronic liver disease	1.21 (0.82–1.79)	0.330

*OR* odds ratio, *CI* confidence interval

## Discussion

In our study, up to one-third of HHC patients developed CAP and required hospitalization; the mortality rate of these patients was 11.6%, which is apparently higher than that in general adults [1]. We further revealed that male sex and nasogastric tube use were associated with a higher risk of hospitalization for pneumonia in the HHC population. Besides chronic diseases, malnutrition appeared to be an important risk factor.

Individuals receiving HHC probably have more comorbidities compared with individuals in the general population. We found that HHC patients with dementia, chronic respiratory disease, or heart failure had an increased risk for pneumonia-related admission. Many studies have recognized chronic respiratory disease and heart failure as risk factors for CAP in adults admitted to the hospital [1, 13, 14]. However, there is no definite conclusion about the relationship between dementia and CAP [3, 15]. A possible reason is that our study focused specifically on HHC patients who might have advanced age and thus a potentially greater risk of severe dementia. Mitchell et al. conducted a prospective cohort study of 323 patients with advanced dementia and found that eating problems and pneumonia were frequent complications, with pneumonia occurring in 41.1% of participants [16]. In addition, our results revealed stroke and chronic liver disease to be significant risk factors in the univariate analysis but not in the multivariate analysis; this is consistent with current evidence: stroke and chronic liver disease have been assessed in a few studies with small sample size, and the association with CAP remains inconclusive [3, 17–19].

The risk for aspiration and aspiration pneumonia with nasogastric tube feeding has been extensively studied, especially in critically ill patients and residents of nursing homes [20, 21]. Differentiating aspiration pneumonia from other pneumonia is difficult. Assessments of aspiration, such as videofluoroscopy and swallowing tests, are neither universally available nor suitable for all the patients. Hence, we did not explicitly distinguish aspiration pneumonia in our study; nonetheless, we surmise that it may frequently occur in this special population. High gastric residue has been considered a risk factor for aspiration in tube-fed patients [22]; however, continuous pump feeding is not better than bolus feeding. Therefore, increasing the frequency and reducing the volume of each feeding are suggested [23, 24].

In previous studies, poor nutrition encompassed conditions such as hypoalbuminemia, anemia, hypoproteinemia, low nutritional score, low BMI, or unintentional weight loss. Nevertheless, despite the lack of a standardized definition, malnutrition assessed according to different criteria was an established risk factor for CAP [3, 25–29]. Our results demonstrated that HHC patients with anemia and hypoalbuminemia have an increased risk of hospitalization for CAP. However, the relationship between BMI and pneumonia was not statistically significant. A possible explanation may be that our study calculated BMI from estimated weight and height. Furthermore, BMI may be unsuitable for elderly because body composition changes with aging [30]. Unfortunately, there is not a single parameter that accurately reflects the actual nutritional condition. Hence, more comprehensive nutritional assessment and research are required for this group.

Interestingly, previous studies identified smoking as an important risk factor for pneumonia [3, 13, 14], but it did not reach statistical significance in our analysis. This discrepancy may be related to a higher proportion of female patients in our study. In Taiwan, the smoking rate is only about 4% among women, but nearly 30% among men [31]. A Cochrane review showed influenza vaccine can reduce the risk of influenza infection in older adults; however, the data in this review were underpowered to detect differences for pneumonia. Therefore, the role of influenza vaccine as a protective factor for CAP remains unclear [3, 32, 33]. The rate of influenza vaccinations was disappointingly low in our HHC population, and prior vaccination did not significantly decrease the risk of hospitalization for CAP. In Taiwan, pneumococcal polysaccharide vaccine is provided free to the elderly aged ≥ 75 years; however, some patients may pay for self-paid vaccine before age 75 years. Additionally, some patients may get pneumococcal conjugate vaccine. Hence, it was difficult to trace whether our HHC patients had received pneumococcal vaccination. Accordingly, further research is needed to evaluate the impact of influenza vaccine and pneumococcal vaccine on pneumonia in this group.

One study also indicated that the infection rate was greater in HHC patients with excessive polypharmacy (taking ≥ 10 drugs) than in those who were taking fewer drugs; however, the study did not point out the specific type of infection [6]. Our findings discovered a higher proportion of excessive polypharmacy in the hospitalization group, but the significance was not found in the multivariate analysis. This may be because we only documented the amount of medications from our hospital, and a detailed classification of drugs or prescriptions from other medical institution was not recorded. In addition, there is no consensus concerning the cutoff point or a strict definition for polypharmacy [12]. Thus, further research needs to consider not only the amount of drugs, but also potentially inappropriate medications.

To the authors’ knowledge, this is the first study to investigate risk factors specifically for pneumonia requiring hospitalization among the general HHC population. However, the study has several limitations. First, this is a cross-sectional study; therefore, the actual cause-and-effect relationship needs further clarification. Second, the true incidence of pneumonia is probably underestimated, because some cases may have been treated in the outpatient setting or patients may have been admitted to another hospital. Although these patients were not included in our study, we surmise they occupied only a small percentage of the all analyses. One possible reason is that most our HHC patients were bedridden, and it was very inconvenient for the family to bring them to the outpatient department. Our HHC patients would like to give priority to our hospital because most of their medical records were in our hospital. Due to old ages and multiple diseases, most of HHC patients required hospitalization if pneumonia was diagnosed in the emergency department. Nevertheless, these are still our limitations. Further outpatient setting study or cross-hospital study is needed. In the end, there may be residual and unmeasured confounding that may not be completely documented in the medical records.

## Conclusions

In HHC patients, pneumonia is a common cause of hospitalization and has high mortality and readmission rates. Herein, we have highlighted the risk factors related to hospitalization for CAP in this special group. Although these findings may be also reported from other populations, it still provides following meaningful information. First, in addition to male sex and chronic diseases, malnutrition is a risk factor that is potentially modifiable. Second, once patients need nasogastric tube use, to reduce the risk of aspiration and pneumonia, caregivers should carefully check tube position, feeding volume, and rate of administration. It is very important because HHC patients are cared by informal care workers and their family. The current study provides important information to create preventive interventions for pneumonia in the HHC population.

## Data Availability

The data supporting the findings of this manuscript can be obtained from the corresponding author on reasonable request.
